# Screening for Hypertension in Children With and Without Autism Spectrum Disorder

**DOI:** 10.1001/jamanetworkopen.2022.6246

**Published:** 2022-04-06

**Authors:** James T. Nugent, Christine Bakhoum, Lama Ghazi, Jason H. Greenberg

**Affiliations:** 1 Section of Nephrology, Department of Pediatrics, Yale University School of Medicine, New Haven, Connecticut; 2 Clinical and Translational Research Accelerator, Department of Medicine, Yale University School of Medicine, New Haven, Connecticut

## Abstract

This cross-sectional study compares the frequency of hypertension screening at health maintenance visits between children with and without autism.

## Introduction

Adults with autism are at increased risk for hypertension, cardiovascular disease, and death compared with the general population.^1,2^ Although hypertension screening (HS) is recommended for children starting at 3 years of age,^3^ there are barriers to blood pressure (BP) measurement in children with autism, including sensory discomfort^2^ and comorbid conditions requiring attention during well visits.^4^ We compared the frequency of HS at health maintenance visits for children with and without autism.

## Methods

Data for this cross-sectional study were obtained from the National Ambulatory Medical Care Survey and National Hospital Ambulatory Medical Care Survey, annual surveys of ambulatory physicians using multistage sampling to produce nationally representative estimates. We included visits to office-based practices and hospital outpatient departments for patients aged 3 to 21 years from 2002 to 2018 in which physicians identified the major reason for visit as preventive care; visits to emergency departments were excluded.^5^ Autism was defined using *ICD-9 *and *ICD-10* codes or a positive response to the question, “Does patient now have autism?” The primary outcome was whether BP was measured during the visit based on physician documentation. For reference to other chronic diseases, we report the frequency of HS during preventive visits for children with attention-deficit/hyperactivity disorder, asthma, depression, diabetes, cerebral palsy, epilepsy, and obesity. The National Center for Health Statistics Ethics Review Board authorized these surveys with waivers of informed consent. The Yale University institutional review board deemed this study exempt from review. This study followed the STROBE reporting guideline.

Proportions were compared using the χ^2^ test. We used multivariable logistic regression to evaluate the association between autism and HS after adjusting for age, sex, race, year, physician specialty, insurance, number of chronic conditions, diabetes, and obesity. We assessed the association between autism and other preventive services. We defined statistical significance as 2-sided *P* < .05. Data were analyzed accounting for the complex sampling design from October 2021 to March 2022 using Stata/SE, version 17.0.

## Results

Of 44 501 visits, 0.5% (95% CI, 0.4%-0.7%) involved children with autism, with median (IQR) age in years (11 [6-18] vs 12 [6-17]), median (IQR) previous visits in past year (1 [0-2] vs 2 [1-4]), and visits with an obesity diagnosis (4.8% [95% CI, 1.6%-13.7%] vs 4.0% [95% CI, 3.6%-4.5%]) similar for visits with and without an autism diagnosis; visits with autism involved more males (80.8% [95% CI, 69.4%-88.6%] vs 41.7% [40.3%-43.2%]). HS occurred in 55.8% (95% CI, 37.3%-72.8%) of visits with autism vs 75.7% (95% CI, 74.1%-77.2%) without autism (*P* = .02) (Figure). When divided into 2 periods, HS increased over time for children without autism but not for children with autism. HS for children with other conditions occurred at least as often as for children without that condition. Among visits with BP measured, hypertensive readings^3^ occurred in 16.7% (95% CI, 7.9-31.8) with autism and 15.2% (95% CI, 14.3-16.2) without autism (*P* = .80). In the multivariable model, autism remained significantly associated with decreased odds of HS (odds ratio, 0.39; 95% CI, 0.17-0.89) (Table) and was associated with decreased odds of height measurement, nutrition counseling, and exercise counseling.

**Figure.**
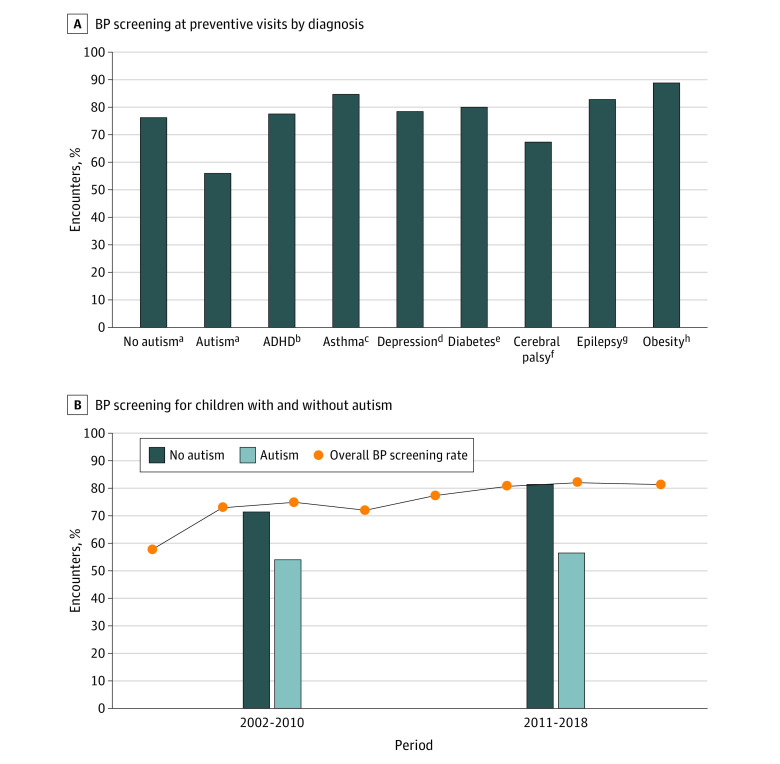
Blood Pressure (BP) Screening During Pediatric Preventive Visits From 2002 to 2018 A, The χ^2^ test was used to compare screening between children with and without each chronic condition. B, For the overall BP screening rate, data were combined into 2-year intervals: 2002-2003, 2004-2005, 2006-2007, 2008-2009, 2010-2011, 2012-2013, 2014-2015, 2016-2018 (no survey data were available for 2017). ADHD indicates attention-deficit/hyperactivity disorder*.* ^a^Autism was defined as *ICD*-*9* and *ICD-10* codes F84.0, F84.1, F84.5, F84.8, F84.9, 299.00, 299.01, 299.80, 299.81, 299.90, or 299.91 or a positive response to the question, “Does patient now have autism?” Thresholds for hypertensive readings were defined according to the 2017 American Academy of Pediatrics hypertension guideline, with median height for age and sex used in cases of missing height. ^b^Defined as *ICD*-*9* and *ICD-10* codes F90.0, F90.1, F90.2, F90.8, F90.9, 314.00, or 314.01 or a positive response to the question, “Does patient now have attention deficit disorder or attention-deficit/hyperactivity disorder?” ^c^Defined as *ICD*-*9* and *ICD-10* codes J45 or 493 or a positive response to the question, “Does patient now have asthma?” ^d^Defined as *ICD*-*9* and *ICD-10* codes F32, F33, 296.2, 296.3, or 311 or a positive response to the question, “Does patient now have depression?” ^e^Defined as *ICD*-*9* and *ICD-10* codes E08, E09, E10, E11, E13, 249, or 250 or a positive response to the question, “Does patient now have diabetes mellitus?” ^f^Defined as *ICD*-*9* and *ICD-10* codes G80 or 343. ^g^Defined as *ICD*-*9* and *ICD-10* codes G40 or 345. ^h^Defined as *ICD*-*9* and *ICD-10* codes 278.00, 278.01, E66.0, E66.01, E66.09, E66.1, E66.2, E66.8, or E66.9 or a positive response to the question, “Does patient now have obesity?”

**Table.** Frequency of Hypertension Screening and Provision of Other Health Maintenance Services During Preventive Visits for Children With and Without Autism From 2002 to 2018

	Visits, % (95% CI)^a^		*P* value^d^	Odds ratio (95% CI)^b^		*P* value
	Children with autism (n = 214)^c^	Children without autism (n = 44 287)^c^		Crude	Adjusted^e^	
Hypertension screening						
Visit type						
Preventive	55.8 (37.3-72.8)	75.7 (74.1-77.2)	.02	0.41 (0.19-0.86)	0.39 (0.17-0.89)	.03
Nonpreventive^f^	36.6 (29.1-44.8)	37.8 (36.5-39.1)	.77	0.95 (0.68-1.33)	1.08 (0.72-1.62)	.71
All	39.8 (32.1-48.1)	47.2 (46.0-48.4)	.08	0.74 (0.53-1.03)	0.96 (0.63-1.45)	.85
Other health maintenance services during preventive visits						
Measured						
Weight	72.8 (46.1-89.4)	88.5 (87.1-89.8)	.06	0.35 (0.11-1.10)	0.40 (0.15-1.03)	.06
Height	64.0 (41.3-81.7)	78.1 (76.3-79.7)	.14	0.50 (0.20-1.27)	0.42 (0.20-0.88)	.02
Temperature	41.1 (25.5-58.7)	40.8 (38.3-43.3)	.97	1.01 (0.50-2.07)	0.88 (0.47-1.64)	.68
Health education provided	58.7 (36.5-77.9)	56.4 (53.3-59.4)	.61	1.05 (0.42-2.61)	0.87 (0.36-2.11)	.75
Dietary counseling provided	20.4 (11.6-33.5)	31.1 (29.1-33.2)	.10	0.57 (0.29-1.12)	0.53 (0.28-0.98)	.04
Exercise counseling provided	10.2 (4.7-20.7)	19.7 (17.9-21.5)	.07	0.47 (0.20-1.07)	0.44 (0.20-0.96)	.04
Depression screening^g^	0.9 (0.1-6.3)	3.7 (2.9-4.6)	.13	0.24 (0.03-1.80)	0.13 (0.01-1.11)	.06
Influenza vaccine administered^h^	9.8 (3.9-22.6)	13.3 (11.4-15.4)	.50	0.71 (0.27-1.91)	0.65 (0.24-1.75)	.39
Cholesterol screening ordered^i^	10.0 (2.0-37.7)	8.3 (6.0-11.3)	.82	1.22 (0.22-6.93)	1.26 (0.28-5.54)	.76

^a^ Percentages are column percentages.

^b^ Odds ratios are for the association between autism and receipt of each preventive service.

^c^ N value reflects the number of preventive visits.

^d^ Proportions were compared using the χ^2^ test.

^e^ Adjusted for age, sex, race (recorded by the physician as Black, White, or other [American Indian or Alaska Native, Asian, and Native Hawaiian or Pacific Islander]), survey year, physician type (primary care or specialty physician), total number of chronic conditions, obesity diagnosis, diabetes diagnosis, and insurance (public, private, other, or unknown). Race was imputed by the National Center for Health Statistics for 25.9% of observations in the sample. All other covariates had complete data except insurance type (18.3% missing) and total number of chronic conditions (18.6% missing). Visits with missing insurance type were coded as unknown insurance. Visits in which the list of chronic conditions were left blank were coded as having 0 chronic conditions.

^f^ There were 1576 nonpreventive visits for children with autism and 127 538 nonpreventive visits for children without autism.

^g^ Depression screening was considered to be indicated for patients 12 years or older.

^h^ Influenza vaccine was considered to be indicated if the visit occurred between October and May. Influenza vaccination was identified by Multum classification code D01164 in any of the medication fields for survey years starting in 2006.

^i^ Cholesterol screening was considered to be indicated if (1) the patient had not had cholesterol levels measured within the past year (based on survey question) and had obesity in any of the survey years or (2) the patient had not had cholesterol levels measured within the past year and was aged 9 to 11 years or 17 to 21 years in survey years after 2011 to reflect changing clinical practice guidelines.

## Discussion

In this national sample of pediatric preventive visits, HS was less likely to occur during visits for children with autism vs visits for children without autism. Our findings complement literature describing gaps in preventive care for children with autism, including decreased vaccination rates and missed well-child visits.^6^ Home BP monitoring may be a feasible alternative to in-office measurement for children with autism.^2^

Limitations include the cross-sectional design, lack of previous BP values, and small sample of visits with an autism diagnosis. Defining autism by physician documentation may introduce misclassification bias, with more severely affected children being more likely to be identified; however, we used broad *ICD-9* and *ICD-10* criteria to capture a spectrum of autism phenotypes. Further research is needed to identify barriers and test interventions to improve HS and cardiovascular disease prevention in children with autism.
